# Cultivated invisibility and migrants’ experiences of homelessness during the COVID-19 pandemic

**DOI:** 10.1177/00380261221100359

**Published:** 2023-01

**Authors:** Simon Stewart, Charlotte Sanders

**Affiliations:** University of Portsmouth, UK; SOAS University of London, UK

**Keywords:** COVID-19, cultivated invisibility, homelessness, migrant, pandemic

## Abstract

The UK government’s Everyone In scheme, announced in March 2020, required local authorities to temporarily house *all* homeless individuals in their area regardless of immigration status. In providing support through safe and secure accommodation, Everyone In also provided a crucial moment of visibility for migrants experiencing homelessness. Yet, just as it provided life-changing opportunities for some, the scheme was not straightforwardly a celebratory moment for migrants. It remained embedded within a wider context of immigration governance and social inequality in the UK, which has both invisibilised migrant homelessness as a crisis and hypervisibilised migrants as undeserving, suspicious or ‘illegal’ subjects. In this article, we explore life-story narratives co-produced with migrants across three urban contexts that capture their experiences of homelessness before and during the pandemic. In doing so, we introduce the notion of *cultivated invisibility*, referring to a habitual, deeply-ingrained mode of practice through which migrants respond to and navigate their experiences of being read as ‘Other’, in racialised or classed terms. It is developed through conditions of material scarcity and in the course of multiple engagements with racial capitalism’s various ‘faces of the state’ in an increasingly hostile environment for migrants. Cultivated invisibility involves staying on the move and blending into the crowd or avoiding it altogether but it also includes the experience of being unseen despite having come forward for help. Importantly, we demonstrate that cultivated invisibility becomes a cause of illegalisation, just as much as a response to it.

## Introduction

Somewhat paradoxically, COVID-19 brought to many migrants experiencing homelessness in the UK the life-changing benefit of unprecedented access to statutory support. This was previously inaccessible due to both various immigration policies which have blocked migrants from accessing welfare services and, for migrants who were eligible for support, various practical constraints ranging from a lack of knowledge of services available to them and racialised experiences of dismissal on the part of local authorities. The UK government’s Everyone In scheme (Gov.uk, 2020), announced in March 2020, required local authorities to temporarily house *all* homeless individuals in their area regardless of immigration status and, in doing so, also increased funding for homelessness support that extended available accommodation options dramatically. In providing support through safe and secure accommodation, Everyone In also provided a crucial moment of visibility for migrants experiencing homelessness and for migrant homelessness more broadly, as many migrants encountered homelessness organisations for the very first time, and vice versa.

Yet, just as it provided life-changing opportunities for some, Everyone In was not straightforwardly a celebratory moment for migrants. It remained embedded within a wider context of immigration governance and social inequality in the UK, which has both invisibilised migrant homelessness as a crisis and hypervisibilised migrants as undeserving, suspicious or ‘illegal’ subjects. The introduction of ‘Hostile Environment’ measures in 2012 required support services to assess the in/eligibility of service users and led to data sharing in relation to ‘illegal’ immigrants rough sleeping (Corporate Watch, 2017), making fear and suspicion of statutory support common amongst migrants, whether ‘illegal’ or otherwise. Additionally, these measures have resulted in an increasingly hostile social environment for migrants in the UK, intensifying racialised vulnerabilities in everyday encounters. Austerity measures have also exacerbated an already palpable housing crisis, which has led both to a crisis in homelessness itself and homelessness assistance, with decreased funding for emergency accommodation and a lack of social housing options.^1^

Introducing *cultivated invisibility* to capture the multiple and intersectional ways in which migrants have internalised invisibilisation as an embodied disposition *and* learnt to survive without statutory support, this article explores enduring experiences of migrant invisibility throughout the pandemic despite the supposedly ‘visibilising’ force of the Everyone In response. As we show, for numerous migrants, experiences of Everyone In reaffirmed a distrust of homelessness support. Rather than approaching the pandemic as a ‘crisis’ in the sense of a break in the normal, then, we consider it as ‘an amplification of something [already] in the works’ (Berlant, 2011, p. 10; see also Sanders, 2020). As such, we argue that Everyone In provides a key moment through which to conceptualise the complex politics of in/visibility as experienced by homeless migrants more broadly, a politics which emerges across and between issues of access, discomfort, suspicion and surveillance, and which illuminates the interrelation of immigration status, racialisation and class in the formation of migrant subjectivities regarding their exposure to harm. Finally, building upon analyses of invisibility in relation to illegality, we suggest that invisibility is not just an *effect* of illegality, but actively produces the conditions of illegality itself. Here, our work is informed by De Genova’s (2002) work on deportability that moves beyond deportation as an event to interrogating the processes that produce migrant subjectivities in relation to the possibility of deportation. We are also inspired by De Genova’s refusal to separate immigration governance from the imperatives of capitalism. As we demonstrate, cultivated invisibility is an adaptive response to both austerity and the hostile environment, as well as a practice that increases the vulnerability of migrants to destitution, homelessness and hostile environment measures.

## Migrant homelessness: The crisis before the crisis

When COVID-19 reached the UK in early 2020, homeless migrants across the country were already in a state of crisis (Sanders, 2020). The numbers of migrants experiencing or at risk of homelessness were both stark and increasing, yet migrant homelessness as a crisis remained largely invisible (Crisis, 2019). One consequence of the invisibility of migrant homelessness in the UK is also its relative absence from sustained scholarly study; although there is significant work on destitution among asylum seekers and refugees (Allsopp et al., 2014; Dwyer & Brown, 2008; see also Galbraith, 2019). Our research responds to this innovatively through multi-sited analysis across three urban contexts, deploying a life-story narrative approach alongside ethnographic research in homelessness organisations. As we show, this enables richly detailed individual accounts of homelessness amongst migrants, which capture the complex and intersectional dynamics of invisibility – and, indeed (hyper)visibility – from the level of the everyday.

Unlike British citizens, migrants – who inhabit a wide range of immigration profiles – are frequently impacted by ‘No Recourse to Public Funds’ (NRPF) conditions,^2^ which block them from multiple forms of statutory support including homelessness assistance. For EU migrants (prior to the end of the Brexit transitional period) eligibility for statutory support has become increasingly stringent in the years since 2013, when the government introduced measures targeted explicitly at ‘restrict(ing) access to benefits for migrants from the European Economic Area (EEA)’ (Gov.uk, 2017, p. 3). Furthermore, for migrants who are eligible for support, numerous challenges remain. First, a lack of knowledge surrounding incredibly complex in/eligibility criteria impacts both the awareness of migrants as to the services available to them, *and* the awareness of frontline staff in local authorities and homelessness organisations. In addition, a housing shortage has increased waiting times for accommodation and competition in who is prioritised for housing, which is in turn impacted by racialised experiences of dismissal in migrants’ encounters with staff.

These realities, in tandem with the classed positionalities that many migrants occupy within precarious and/or informal economies, make migrants in the UK extremely vulnerable to homelessness, and also make it exceptionally difficult for them to overcome homelessness. Part of the work of our research is to provide a way to account for the complex interrelations of these dynamics in shaping, as Pugh underscores, ‘the ability (or not) to access in practice the formal protections and rights that are guaranteed in formal law’ (2021, p. 1). This is particularly important in relation to conceptualising invisibility, as accounts of migrant invisibility tend to focus on the experiences of ‘undocumented’, ‘illegal’, or ‘irregular’ migrants (Humphris & Sigona, 2019; Kukreja, 2021; Mazzara, 2015; Villegas, 2010). Alongside the risk of ‘methodological nationalism’ (Wimmer & Schiller, 2002) in setting the parameters of research subjects around categorisations provided by state immigration apparatuses, this also invisibilises the experiences of migrants who are ‘documented’, ‘legal’ or ‘regular’, as well as hindering the capacity to conceptualise dis/continuities across migrant experiences of invisibility. Here, our research strives toward a conceptualisation of invisibility as emerging through the intersections of immigration status, racialisation and class, seeing invisibility as a way to diagnose power beyond binaries of un/documented, il/legal and ir/regular. Indeed, as some of our participants had become British citizens, this also pushes back against a citizen/non-citizen dichotomy in studying migrant invisibility. This approach moves beyond migranthood as exceptional, toward studying migration as a means to conceptualise social categories and inequalities, and their intersectional dynamics and effects, more broadly.

Similarly, accounts of migrant invisibility have often analysed invisibility as a *strategy* pursued by – again, ‘irregular’, ‘undocumented’, ‘illegal’ – migrants as a way to mitigate the risk of deportation or challenge exclusion (Caraus, 2018; Villegas, 2010; Wahlström Smith; 2018). Our aims diverge from this approach of conceptualising invisibility as an effect of illegality; indeed, again, many of our participants were not irregular migrants (although some were), and had not arrived in the UK ‘illegally’ either, and yet invisibility was a continuous experience across the life-story narratives we gathered. Instead, what we demonstrate is that invisibility is cultivated through intersectional experiences of alterity, which include but are not limited to immigration status. Furthermore, we show that cultivated invisibility actually produces illegality and irregularity. As such, then, invisibility is both a response to illegality, and is itself productive of it. This is particularly pertinent in a contemporary moment when the UK government has just introduced measures through which rough sleeping will increasingly form the grounds for deportation, underscoring that the invisibilising effects of homelessness also expose migrants to illegalisation (Cromarty, 2020). We argue that Everyone In provides a fruitful moment for complicating analyses of migrant invisibility, conceptualising invisibility as emerging through relational, embodied and habitual experiences (see also Humphris & Sigona, 2019), and not only as a strategic mode of being. We also demonstrate that producing theory that is grounded in life-story narration offers a crucial way to produce richer, more nuanced accounts of migrant invisibility, beyond invisibility as simply an effect of illegalisation.

## Cultivated invisibility: Thinking beyond ‘strategy’

Migration research across multiple disciplines highlights how invisibility is characterised by a power relation between the ‘seer’ and the ‘seen’ (Wahlström Smith, 2018). Exploring migrant ‘“illegality” as lived through a palpable sense of deportability’ (De Genova, 2002, p. 439), with the deportability regime as the ‘seer’, such research shows how undocumented individuals seek to blend into the ‘ordinary’ and position themselves as assimilated so that they remain unseen (Wahlström Smith, 2018). ‘Irregular’ migrants will avoid asking people for directions because of a fear that their irregular status will be uncovered (Sager, 2018). Undocumented migrants will deploy ‘strategic invisibility’, hiding their status by instead assuming the legitimate visibilising identities associated with being a student, a member of a union, or an entrepreneur (Villegas, 2010). Alternatively, self-representation can provide a means of gaining visibility as a form of resistance, as in the case of organised migrant protests. This rich body of literature explores how migrants have to negotiate the potential losses and gains associated with in/visibility within the field of power (Tyler & Marciniak, 2013). In these accounts, in/visibility is often conceptualised as a *strategic* mode of action. In introducing the notion of *cultivated invisibility*, we take a slightly different tack, focusing on the *habitual practices* through which the homeless migrants in our research respond to various experiences of being ‘out of place’, experiences which speak to multiple modes of being read as ‘Other’, both racialised and classed, across local and national space. Cultivated invisibility is deeply rooted in the material conditions of scarcity that migrants have experienced in the hostile environment and the extractive capitalist social relations in which they are situated. Cultivated invisibility is structured by the past and yet is a mode of conduct that orientates the individual’s actions in the present ‘without presupposing a conscious aiming at ends or an express mastery of the operations necessary in order to attain them’ (Bourdieu, 1990, p. 53).

Cultivated invisibility is an embodied and habitual response to and a product of objective life chances which have tended to offer more threats than opportunities, with anxiety and precarity the consequences of the nationalistic (anti-migrant, autochthonic) and neoliberal (pro-market, stripping back of the social state) policies of successive governments (Tyler, 2020; Yuval-Davis et al., 2018). Cultivated invisibility navigates threats and involves staying on the move and blending into the crowd or avoiding it altogether. Such practice can be a means of getting some sleep on a bus or a train and a means of avoiding the stigma associated with poverty, homelessness and migrant ‘Othering’. It also derives from an acute awareness of the potential risks involved in coming into contact with representatives of state or third sector organisations, even among those who have permanent residence. It is a mode of practice that encompasses those who have experienced hostile entanglements with various race-making ‘faces of the state’ (e.g. surveillance, audits, anti-migration politics, NRPF) as well as class-based stigmatisation in service of capitalism’s extractive forms (e.g. through labour precarity, cutbacks to the social state, austerity, affordable housing shortages [Humphris, 2019; Şimşek, 2021; Tyler, 2020]). Our research recognises both continuities and discontinuities across classed and racialised experiences.

Cultivated invisibility does not deny the possibility of consciously-formulated decisions even if these are practical adaptations based on what is possible in the given circumstances. In our research, many migrant participants provided specific details about how they sought to blend into the crowd in order to avoid suspicion and avoid situations that might be threatening. For example, respondents explained how they slept on public transport and adopted the role of commuters on their way to work in order to avoid attracting attention. Our research suggests that through cultivated invisibility, migrants both adapt to life without statutory services and, ultimately, learn to avoid them. Importantly, whilst the cultivated invisibility afforded by ‘passing’ or going unnoticed in space can ensure comfort, safety and survival for urban inhabitants already defined as outside of the dominant hegemonic, this is not to suggest that cultivated invisibility ensures ‘safety’ for migrants. Indeed, it is the invisibility of migrants experiencing homelessness, and migrant homelessness more broadly, that has made homeless migrants particularly vulnerable to harm, ill-health and premature death (Mayblin et al., 2020).

The flipside of cultivated invisibility is the desire to be seen, to come forward, to get help. This, of course, is something that migrants experiencing homelessness frequently (attempt to) do. However, as our research shows, it is difficult to come forward when you have long experienced a sense of being *out of place*, whether in relation to hegemonic notions of respectability (Skeggs, 1997), because of the social stigma of homelessness (Tyler, 2020); or in relation to British citizenship, racialised as outside of the boundaries of the national community and potentially labelled as ‘illegal immigrants’ (Tyler & Marciniak, 2013).

## Cultivated invisibility: Race and racialisation

Whilst migrants experiencing homelessness are clearly not a homogeneous group, they do share – in Simmel’s terms – the role of the stranger who ‘comes today and stays tomorrow’ (Simmel, 1908/1971). Nevertheless, as a social category ‘the homeless’ are de-individualised through the very processes which construct ‘them’ as a group that requires our attention, with key inter- and intra-group differences overlooked (Bourdieu, 2020; Erel, 2010; Erel & Ryan, 2019; Munt, 2016; Stewart, 2013). The experiences of in/visibility that we explore in this article are likely to be shared by migrants and ‘non-migrants’ alike. However, our research underscores the ways in which both visibility and invisibility manifest in racialised ways and particularly in relation to surveillance, in the context of the hostile environment. Researching migrant homelessness disaggregates ‘the homeless’ by underscoring the need to attend to racialisation within varied experiences of homelessness. As Sara Ahmed reminds us, contrary to the assumption that the stranger is the one whom we ‘fail to recognise’ (2000, p. 21), the stranger comes to be marked as such precisely because they are always already recognised and recognisable as out of place as a racialised ‘Other’. To read a body as strange requires knowing what makes a stranger, using techniques of everyday bordering in which experiences of proximity to others involve an assessment of belonging/unbelonging (Sibley, 1995). Whilst all individuals experiencing homelessness may be constructed as ‘out of place’, migrants’ experiences of alterity intersect with racialised assessments of ‘Otherness’. Given the diffusion of state power, these assessments are conducted not only by bureaucrats in government departments but also by various ‘faces of the state’, including volunteers working for charities and churches (Humphris, 2019, p. 107).

The need to recognise strangers emerges through a hegemonic concern to ensure the safety of the assessing or dominant community. Here, Ahmed cites Elijah Anderson’s following observations of a gentrifying neighbourhood in the city of Philadelphia:

. . . many residents are concerned about the strangers with whom they must share the public space, including *wandering homeless people*, aggressive beggars, muggers, anonymous black youths and drug addicts. (1990, p. 238, cited in Ahmed, 2000, p. 22, our emphasis)

These figures of ‘stranger danger’ remind us that it is particular bodies that come to be recognised as ‘stranger than others’ (Ahmed, 2000, p. 52) and that in their construction as dangerous these figures are always inhabitants of the shadows, of ‘*dark* spaces’ (p. 33, our emphasis). Here, Simone Browne alerts us to the racialised and racialising nature of surveillance, in which ‘surveillance practices, policies and performances concern the production of norms pertaining to race and exercise a “power to define what is in and out of place”’ (2015, p. 16). For Browne, the Foucauldian notion of surveillance as ‘integral to modernity’ must also make explicit that ‘today’s seeing eye is white’ (citing Fiske, 1998, in Browne, 2015, p. 17; see also Davis, 2006).

## Methodology: Researching migrant experiences of homelessness

In this article we introduce data from the findings of a broader ESRC/UKRI-funded research project entitled ‘Homelessness during the COVID-19 Pandemic: Homeless Migrants in a Global Crisis’. The fieldwork for the project spanned 18 months of research in partnership with staff from nine homelessness organisations across three cities in the South of England, and with non-UK national clients engaged by these services throughout the pandemic. In phase one, research with staff (including managers and support workers) was conducted through semi-structured interviews. In phase two, life-story interviews were conducted with migrants. We gathered 37 interviews with staff, and over 100 hours of life-story narrative interviews with 43 migrants, some of whom were receiving homelessness support through the services with which we have partnered. Indeed, it was largely down to the Everyone In initiative that we were able to build connections with migrants who would not previously have been accessing homelessness support for, as we have noted, a wide variety of reasons. In line with the im/possibilities of social distancing measures – which fluctuated throughout – interviews were conducted face-to-face and remotely via video-call and/or audio-call settings. All except two interviews were voice-recorded and transcribed, and where voice-recording was declined, transcripts were produced via interview notes. Most interviews were conducted in English; interpreters were used in 12 cases. At moments when lockdown restrictions were eased, we also conducted ethnographic research with homelessness organisations across our research sites.

As a research parameter, we defined both ‘homeless’ and ‘migrant’ in the broadest possible sense. How ‘homeless migrants’ are defined plays a crucial role in how they are made visible or invisible both socially and politically. Assuming who ‘counts’ as a migrant risks excluding more complex or non-linear experiences of geographic movement and stasis, and relies overmuch on definitions from the state immigration apparatus (again, see Wimmer & Schiller’s analysis of ‘methodological nationalism’, 2002). In our research we considered homeless migrants to include any individuals who are non-UK nationals (including those who now hold British citizenship) and who define themselves as homeless. All of our respondents were born outside of the UK.

We pursued pseudonymous life-story narrative methods for making space for complex personhoods and messy experiences (see also Benson, 2011), acknowledging the particular vulnerability of those racialised as ‘Other’ to anonymising – and dehumanising – accounts (Said, 1978; Spivak, 1988). We brought insight from decolonial methodologies which cite the power of life-story narration in pursuing more participatory, dialogical, caring and reciprocal knowledge production (Denzin et al., 2008; Srigley et al., 2018). The life-story method recognises that the lives and experiences of individuals constitute a forceful, critical contribution to (and intervention within) how the social world is theorised (Brannen, 2019; Harrison, 2008; see also Bruner, 1986). Life-stories can make more visible alternative modes for seeing the world, and situate narrator participants as experts in the contexts and conditions of their own lives. Wherever possible, life-stories were co-produced over multiple meetings, leaving room for contradiction and irresolution in accounts of migration and homelessness. This also provided opportunities for trust-building and, as have others (Etherington, 2009; Kearney, 2007), we recognised the potential for therapeutic benefit in sharing life-story dialogues.

All data were analysed through using thematic analysis (Braun & Clarke, 2006). As an interdisciplinary research team, we pursued a specifically collaborative form of thematic analysis which involved meeting on a regular basis to discuss the data and generate themes (Richards & Hemphill, 2018). Additionally, interviewers produced vignettes of each individual migrant interviewed, and the vignette genre is intended to provide room for the rich texture of individual life stories to be shaped. In the sections below, we draw on these vignettes in order to introduce the individuals whose life stories shaped our following analysis.

In line with the scope of this article, and in order to provide space for more complex intersectional accounts of invisibility, we have chosen to focus on four life-story narratives, those of Albert, Ali, Alexandru and Joshua. These individuals were chosen as reflective of the different circumstances of our participants more broadly: Albert is a German man who had lived in the UK for 46 years with indefinite leave to remain as an ‘EEA migrant’ (until Brexit); Ali is an Afghan man who had held refugee status in the UK for 22 years, with recourse to public funds; Alexandru is a Romanian man who had arrived in the UK four years ago in search of work as an ‘EEA migrant’; and Joshua is an ‘undocumented’ Singaporean man without recourse to public funds, although he had held a regular visa status – as a student – on arrival to the UK 35 years ago. Focusing on these stories enables us to illustrate dis/continuities in migrants’ experiences of visibility beyond ‘illegality’, as well as allowing us precisely the rich detail necessary for our account of invisibility beyond the ‘strategic’. Here, all four ‘case studies’ are based on accounts provided by men. Importantly, and despite our efforts, our research engaged far fewer women than men, indicating the need for further research on the gendered politics of invisibility in relation to migrant homelessness.

## Migrant homelessness in the UK: Intersectional invisibilities

In this section, and through the life stories of Albert and Joshua, we demonstrate the co-constitutive relationship between (cultivated) invisibility and illegality. In both cases, invisibility was not simply an effect of illegality but emerged through a complex interaction between class, racialisation and immigration status, which, in turn, produced the conditions for increasing proximity to illegality. As their stories show, it was in fact job loss which provided a key moment in Albert’s and Joshua’s invisibilisation, as being made redundant led to a loss of housing (also due to the particular relationship between housing law and immigration law), which then in turn led to a lack of access to crucial immigration advice and support. Cultivated invisibility provides a crucial lens of analysis for understanding the intersectional experiences of homeless migrants between austerity, racialisation and immigration governance.

### Albert^3^

Albert is a 71-year-old man, who arrived from Germany in 1975. For many years he worked in hospitality and catering, house-sharing with colleagues who later became friends. As he grew older, Albert became unable to find contemporaries to live with. When opportunities to house share dried up Albert reasoned that because he was working night-shifts at a fast-food restaurant he did not need anywhere to stay, as ‘when other people were sleeping I was working . . . I worked from 12 til 8 continuously. So, I’d get a couple of hours where nobody was there.’ By 2000, when redundancies meant that Albert’s service as a cleaner was no longer required, Albert adapted by shifting to the informal economy. His former manager allowed him to sleep on the premises for a couple of hours in exchange for unwaged labour. Additionally, Albert worked for a market trader for a very small amount of cash-in-hand. He would distribute newspapers, receiving freebies from market traders in return. The loss of formal work made Albert ineligible for benefits because since 2013, hostile environment measures have sought to block EEA migrants from accessing benefits unless they can provide proof of ‘meaningful’ – regular, consistent and taxable – employment (Gov.uk, 2017). This situation was exacerbated when COVID-19 caused the restaurant he was informally working in, and sleeping in, to close, and in 2020 Albert began to sleep on night buses. Eventually, as buses became less populated, Albert was picked up by outreach workers and set up in emergency accommodation through *Everyone In.* Like many migrants who have experienced homelessness, Albert’s ID documents had been lost over the years, hindering his ability to secure ‘settled status’ after Brexit.

Although Albert was homeless from the beginning of 2000 he is unlikely to have featured in any homelessness statistics because he did not seek out statutory support. Instead, Albert worked within the shifting constraints of his life in order to survive. Firstly, it meant ‘blending in’ with the early-morning commuters so as not to appear homeless. Secondly, it involved a habitual mode of vigilance:

I used to make sure that I’d wake up on time, so they wouldn’t have to wake me up. And sometimes when it was a long route and I wanted to change from another bus, I’d get off one or two bus stops too far and then I had to walk back to go where I wanted to go, which was no problem. But anyway, at least I’d make sure that I didn’t go right to the end of the route . . . that I found out is a very good way as well.

Albert practises cultivated invisibility: this habitual, bodily disposition – developed out of necessity and under conditions of scarcity and suspicion – allowed him to move around the city unnoticed, and to catch a few minutes of sleep without attracting attention. However, this practice also took him further in the direction of illegality through informal labour as well as beyond the sight of statutory support and homelessness organisations. As a consequence, he missed the first round of Everyone In. As Albert notes, ‘I was always on the move. It was quite a long time before people realised that I had nowhere to go.’ Albert was particularly vulnerable, as in the absence of support he lived without crucial resources such as ID documents, a bank account and a National Insurance number. Albert was largely unaware of the devastating changes that Brexit would make to his legal right to remain in the UK. Before Everyone In, Albert did not know that he needed to apply to the EU Settlement Scheme (EUSS) before the 30 June 2021 deadline and, either way, would not have known how to access or replace the necessary documents to apply. As we see in this example, Albert’s cultivated invisibility was a means of adaptation to job loss, homelessness and participation in the informal economy. However, as a consequence, he had more exposure to illegality.

### Joshua

Joshua is a 75-year-old man. Already a successful police officer in Singapore, Joshua travelled to the UK in 1986 to undertake a law degree. During his second year, Joshua was informed that his student visa renewal application had been rejected by the Home Office as it was one day late. He appealed and won but only after three years, meaning that Joshua missed his final exams. Joshua was given 21 days to leave the country but won another appeal and stayed. He built a successful life but his immigration status still included a NRPF condition. In 2012, Joshua was made redundant, and losing his job led to losing his accommodation (which he had had for 17 years).

Joshua emphasised his reluctance to intrude on other people’s private spaces. Instead, he chose to sleep on the night buses:

I used to take bus number 25 service – I believe that was the longest service road in the UK, number 25, which used to start from Oxford Street in the West End of London right to the East End of London. So, I used to take the bus about nine or ten at night-time and I would make up and down from one terminal to the other terminal, three or four trips.

In common with many respondents, Joshua found ways to move among the crowd without being observed. This was especially important to him as he was keen to avoid imposing on anyone or to take something to which he was ‘not entitled’. Joshua’s practice of cultivated invisibility was inculcated through having lost his income and place of residence through a brutal work restructuring whilst also being forced into successive lopsided battles with an increasingly hostile Home Office for his right to remain in the UK. Joshua’s story exemplifies how the practice of cultivated invisibility enabled him to adapt to his precarious situation but also rendered him more exposed to illegality.

Somehow, Joshua managed to maintain his stance as an Anglophile, despite being made to feel ‘out of place’. However, he now had to blend in to the crowds to maintain his sense of dignity, and he registered at a gym where he could shower, exercise and wash his clothes. He also managed to get moments of sleep in cafes:

There were days as long as two weeks I never had a sleep, where if at all I slept somewhere maybe on a chair in a cafe or a shop. There were instances I was drinking coffee – that was my sleep.

Years of moving from place to place, finding ways of eating, washing and sleeping by practising cultivated invisibility, took their toll on Joshua’s health, especially as he didn’t have the requisite documents to access healthcare. He lost the sight in one eye, lost most of his teeth and contracted diabetes during his period of homelessness, which lasted until 2018 when he was approached by outreach workers at a bus terminal. Despite numerous setbacks due to his NRPF status, caseworkers were finally able to secure safe and stable accommodation for Joshua. Now, they are working to secure his indefinite leave to remain after 20 years of UK residency.

## Overnight eligibility: The ‘visibilising’ force of Everyone In

In March 2020, the UK Government announced the Everyone In scheme in England (Gov.uk, 2020). Research, including our own, suggests that one of the most impactful consequences of the scheme was how it made migrants (or ‘non-UK nationals’) such as Albert, Joshua, Ali and Alexandru visible to homelessness services, producing invaluable connections to support (see also Coombs & Gray, 2020; Dickson et al., 2020; National Audit Office [NAO], 2021). As one support worker explained:

[What] the scheme has done during the pandemic has increased the visibility of a lot of people that are rough sleeping who might be missed or not be assessed or might not be able to seek support. So, I know that we’ve seen a big increase in terms of the people that we are seeing and able to make referrals for. (Jane, homelessness support worker)

In providing access to support services, the scheme also made welfare assistance visible to those migrants who may have previously been eligible but unaware of their entitlements:

A lot of our clients just don’t know that, [they] have no understanding of their benefits and entitlements . . . [they] just don’t know what their entitlements are and how to access them. And yes, that’s why they end up homeless. Almost no European national, until we tell them or support them with that, have known that they’re entitled to benefits, a lot of them don’t even know what benefits are. (Jay, homelessness support worker specialising in EU casework)

In this section, Ali and Alexandru elucidate the potentially life-changing consequences of Everyone In. In providing access to emergency accommodation for *all* individuals experiencing homelessness, Everyone In intervened crucially in the invisibility cultivated through intersectional experiences of racialisation, immigration governance, exploitative working conditions and the housing crisis. Everyone In provided Ali with some respite from the racialised politics of access to accommodation. Meanwhile, it saved Alexandru from potential deportation by enabling access to immigration advice.

### Ali

Ali is a 41-year-old man from Afghanistan, with refugee status granted 22 years ago. Ali was not offered mental health support during his asylum process, despite struggling with symptoms of Post-Traumatic Stress Disorder (PTSD). Ali’s mental health led to risky patterns of behaviour, including driving under the influence. This resulted in a criminal record and prison time, blocking Ali from securing employment, stable housing (in the form of his own tenancy) and citizenship rights in the years since, as access to all of these are hindered by recorded criminal convictions. For some years Ali successfully sofa-surfed, drawing on a solid friendship network. When options dried up in 2018, he began sleeping in his car. Two years of this led to extreme weight loss and skin and joint problems. Finally, Ali’s GP signposted him to a local homelessness support organisation, and he was eventually accommodated through Everyone In. Ali’s wife and children continue to live in a refugee camp in Pakistan.

Ali’s experiences of cultivated invisibility centred on multiple failed attempts of seeking support from his Local Authority, support that Ali was entitled to as a refugee. Ali recalls feeling repeatedly ignored by Local Authority caseworkers, who he felt were minimising his situation:

[They would say], ‘there’s plenty of people sleeping in the town. So, it’s not only you . . . there’s plenty more there’. Then my mind was saying, no, I have PTSD, I’ve got a problem. ‘Oh, lots of people have the same problem’. Everything I tried to excuse to them, and they would try to just push me back.

In the context of a housing crisis, accommodation options are increasingly scarce. Yet, Ali also felt that local authority housing officers were particularly dismissive of him because he was a refugee, and he situated these encounters within a wider context of racialised and racist encounters with the police, the Home Office, the Job Centre and the general public. After several attempts, Ali gave up on seeking support and worked instead to keep himself safe by sleeping in his car, parked up in a local car park which he knew to be empty at night. Cultivated invisibility meant that Ali was not picked up by homelessness outreach workers, and it was instead his doctor who made him aware of local support services, after which he was housed through *Everyone In.* Ali’s emergency accommodation provided him with a safe place to stay and washing facilities. Prior to the pandemic, hygiene was a huge issue for Ali:

Nobody can give you anything if you be sleeping rough. You’ve got no shaving, you’ve got no clean shoes, you’ve got no clean you, didn’t have a shower for three months, four months, so I don’t think that somebody will interested to give you accommodation, forget about a one bedroom flat.

For Ali, Everyone In has been most centrally an experience of becoming visible. Alongside emergency accommodation, the scheme introduced Ali to support workers who could advocate on his behalf. In shining a light on the sheer volume of individuals vulnerable to homelessness in the UK, Everyone In has also led to increased funding for local homelessness responses that is translating into long-term housing solutions for people like Ali. Ali’s caseworker explored his particular circumstances and liaised with the Local Authority’s housing team on his behalf. After a short time in emergency accommodation, Ali was set up with an affordable room of his own that he can stay in indefinitely, paid for monthly by statutory housing benefit.

For Ali, this was finally a place to sleep that felt ‘like home’, and which provided a good balance between feeling supported and feeling independent. As Ali explains, ‘they can clean your room, they can do everything for you, you can have a breakfast, you can have tea or coffee’. For Ali this is incredibly useful, as his mental health issues can sometimes make self-care difficult. Nevertheless, that the room is not in a purpose-built support block means that Ali has ‘no restrictions’ on his movements and (unlike in a hostel) he can ‘go anytime out’ and ‘come anytime inside’. That his accommodation is quiet allows Ali to manage his PTSD symptoms; its ground floor location also helps with his lower back problems. For Ali, it was this feeling of being seen – both as homeless *and* as an individual with a particular life history – that was the most life-changing consequence of Everyone In.

### Alexandru

Alexandru is a 40-year-old man from Romania, who arrived in 2017. This being just one trip of many across his adult life, Alexandru did not plan to stay long-term but rather intended to work here temporarily. In the UK, Alexandru’s employment experiences were exploitative. All of his employers paid cash in hand, not making him aware of requirements like National Insurance numbers or taxation. When work opportunities vanished, Alexandru was blocked from claiming welfare support as he was unable to evidence his employment and, therefore, could not pass the ‘Habitual Residence Test’ (Gov.uk, 2017) or his Local Authority’s homelessness support eligibility assessment. Alexandru was forced into rough sleeping, a situation which continued until Everyone In.

In suspending eligibility criteria for support, Everyone In led to a significant paradox for homeless migrants: namely, that whilst the pandemic caused a moment of crisis and insecurity it also provided (for some) respite and safety. For Alexandru, aspects of the pandemic were, in fact, ‘lovely’, not least that it was the first time he had felt safe after years of sharing precarious accommodation or rough sleeping. Alexandru felt strongly that easy access to food had been the biggest improvement in his everyday life. Having lived for so long without access to and/or knowledge of support, Alexandru had relied upon begging in order to eat, a traumatic experience that he still struggles to talk about. Aside from meals provided by the Local Authority, the encounter with homelessness services also meant a new wealth of knowledge regarding wider support available locally, including church provisions and foodbanks. This knowledge will last well beyond the pandemic, providing a previously unknowable safety net. Church groups have also led to social connections, including a friend who keeps Alexandru’s phone topped up and who gave him the money to secure his National Insurance number.

Similarly to Albert, when Alexandru lost his travel documents on a night out he had no idea of how to replace them. Alexandru also had no knowledge of the EU Settlement Scheme, or of the fact that without settled status he would eventually be forced to leave the UK after Brexit. As he explains:

The bottom line is that I didn’t have a clue of what my rights were when I came to this country and what to do.

I: When did that change?

When I started mingling with people and being here.

In making him visible as homeless, the Everyone In scheme included Alexandru in a network of resources, including a wider community of individuals with experience of homelessness and of service providers. These invaluable sources of information helped Alexandru to meaningfully approach his situation for the first time. Due to the scheme Alexandru was able to secure settled status, alongside knowledge of employment standards, welfare entitlements and how to access them. He now has permanent work, and a secure tenancy of his own.

## The limits of visibility: Old and new experiences of invisibility in the COVID-19 response

In this section we highlight how cultivated invisibility endures over time and also how new experiences (such as Everyone In) reinforce it. For Albert, it remained ingrained in his body; for Alexandru, it remained embedded in his interactions with service providers.

### Albert

When Everyone In was launched in March 2020, Albert was not immediately identified as in need of help. However, his cultivated invisibility lost its effectiveness in early 2021 when the icy weather set in. The national lockdown at that time meant there were fewer people on the buses and largely deserted train stations made their lack of a home more visible to outreach workers and the state:

I: So, was it just [because of] the cold spell that people saw that you were needing a place or . . . how do you think you were spotted then?

I don’t know! It’s a guess really. Probably what made it worse was because it was restricted and you were not allowed to travel anyway, at this time. Probably, 95% of people at the station at this time were homeless anyway, so I think that’s why it happened.

Cultivated invisibility means that many migrants are likely to have missed out on at least some of the benefits associated with the Everyone In initiative. Albert’s cultivated invisibility also came with side-effects when he finally had a place to stay in the hotels. He had developed a deep-set inability to sleep beyond the duration of an average bus journey. He woke up every hour; this hyper-vigilance was inscribed in his bodily disposition as an enduring effect of invisibility, cultivated over the longue durée of his vulnerability:

Yea so I’ve got this place now. I’ve been here about three months, and I thought things would change. But I’ve found that every night I wake up and I look at my watch and it’s been half an hour or 55 minutes or 1 hour and 5 minutes. Every single night I wake up, not because it’s noisy or anything like that, I just wake up. I go 11 o’clock to bed, I go to sleep and then I wake up and I look at my watch, oh, 12 o’clock. So, I think of something, turn off the light and go back to sleep again until I wake up again. It’s like a dream, it’s like an episode, until you wake up and you see oh it’s only been an hour again. But every night, every night is the same. And I don’t need an alarm clock, because like today I wake up 4 o’clock, wake up 5 o’clock, wake up six o’clock no problem. If I have to wake up at four o’clock I get up, if not I turn back round to sleep, five o’clock I wake up again.

Whilst the scheme had somewhat interrupted Albert’s cultivated invisibility, a long-term process of invisibilisation as a result of job loss, destitution (also shaped by immigration governance) and homelessness remained inscribed on his bodily rhythms.

### Alexandru

For Alexandru, invisibility remained a core concern throughout the crisis. Alexandru described feeling unseen within the homelessness service charged with his care:

I feel that they always put my requests at the bottom of the list . . . I’m not complaining that they don’t help. It’s just that they never seem to prioritise [me].

In this example, we see that Alexandru’s cultivated invisibility is a product of his interaction with support staff, which leaves him feeling unseen and unvalued. As such, invisibility is reinforced in embodied, interactional and habitual ways even as Alexandru’s invisibility was supposedly interrupted by the Everyone In initiative. Here, Everyone In provides a crucial moment to understand the invisibilising effects of encounters with statutory services more broadly, encounters that are productive of migrant distrust and avoidance of services in ways that cut across ‘irregular’ and ‘regular’ migrant categorisations.

Whilst Alexandru felt that he was treated differently as a Romanian, he also demonstrated awareness that support workers were constrained by their heavy workload. Alexandru’s support worker consolidated this point, underscoring that it was her two ‘non-UK national’ clients who had the most intricate and overwhelming cases. Her struggle to address their problems derived in large part from a lack of training for homelessness support workers in immigration matters:

I mean, I was given these two clients and told I had to try and sort out their settled status and this and this and this. You may as well have been talking in a foreign language, ’cause it didn’t mean anything to me. (Kathy, homelessness support worker)

Just as Everyone In provided an opportunity for previously ineligible migrants to access homelessness support, services were not always prepared for migrants’ particular needs, having little experience with the interplay between immigration policies and housing law. Immigration advice was particularly important for Alexandru because he was trying to understand his situation post-Brexit:

Okay. So, I don’t think that I understand Brexit even now. So, I am not sure that I understand properly Brexit now. What I understand more now is that Brexit is not about foreigners leaving the country. It’s about people paying taxes.

Alexandru struggled to access information about the impact of Brexit on his legal right to remain in the UK. Without the requisite immigration training and access to intepreters, Alexandru’s support worker was left to rely on Google in her attempts to understand the EUSS:

No, I mean, I didn’t understand, even when I was given the case, ’cause I’d never worked with any migrants before. I didn’t know the difference between pre-settled and settled status. I didn’t know they had to apply for this. I didn’t know any of this. So, I’ve had to learn and try and do the best I can for him, all at the same time.

Alexandru’s support worker was also painfully aware of the effect of this upon him:

It must be terrifying . . . Being him. ’Cause he knows. He knows . . . He’s got a calendar in his room with the date on.

In common with others interviewed, Alexandru felt a high degree of uncertainty about the future, and fear that he would be invisibilised again when the COVID response ended. Indeed, this was compounded by the temporary experience of visibility – shelter, food and clothing – that Everyone In made possible. In addition, Alexandru’s story highlights precisely the ways that invisibility, cultivated across multiple settings and encounters, has an illegalising effect. Without Everyone In, Alexandru would not have lodged his EUSS application, exposing him to deportation post-Brexit. At the same time, the invisibility of migrants within statutory services has also resulted in an absence of immigration knowledge amongst support workers themselves, hindering their ability to provide effective help.

## Invisibilising experiences of hypervisibility during COVID-19

Whilst Everyone In provided opportunities for visibility, it also set in motion modes of visibility which felt uncomfortable, intimidating, and sometimes frightening. Just as unprecedented inclusion within a local and national homelessness response provided crucial resources for overcoming homelessness, new proximity to statutory services also engendered fresh modes of state regulation and surveillance (Butchinsky, 2017; Johnsen, 2016). Ali’s and Joshua’s stories highlight how their experiences of being observed in the accommodation provided led to compounded feelings of exposure and enclosure within a context of multiple marginalisation.

Surveillance is a racialised technology of discipline in which the state and experience of being observed increase and deepen with one’s perceived distance from normative whiteness (Browne, 2015; see also Ahmed, 2000; Davis, 2006). Indeed, marked as troubled or ‘revolting’ subjects (Tyler, 2013), individuals experiencing homelessness inhabit a symbolic distance from hegemonic whiteness and middle-class normativity, a social abjection which provokes multiple modes of observation: from rough sleep counts, to police monitoring, to surveillance within support services themselves. Being racialised as ‘migrant’ exacerbates this subject position. Hypervisibilised by these various forms of surveillance, without feeling that their voices were being heard, Ali and Joshua felt stigmatised as ‘matter out of place’. These uncomfortable experiences that resulted from coming forward for help illuminate the reasons why cultivated invisibility is necessary in the first place.

### Ali

Just as Ali’s experience of sleeping in his car was marked by fear and insecurity, his experience of the emergency accommodation provided by Everyone In was not simply one of safety and security. The local government response to the scheme provoked multiple forms of surveillance in emergency accommodation blocks which rubbed up against the particular context of Ali’s life, in which symptoms of PTSD have been a long-term struggle. In the apartment block in which Ali was living, surveillance mechanisms included security guards monitoring the doors in and out of the accommodation, and ‘welfare’ checks throughout the day. As Ali describes:

[You have to] put your name in here, and then, someone’s sitting at the door, to say, you know, I’m this, room number 38 . . . and they wake you every day from sleep, like, I mentioned to you, if somebody has a mental health issue, it’s not a suitable place to be lived at, because if you go to sleep, and they’ll be knocking your door, saying wake up, that would mean your brain would be disturbing.

For Ali, the frequent sound of door-knocking – whether his own door or those of his neighbours – triggered his PTSD symptoms, and the ability of staff to enter his room upon him not answering jeopardised his feelings of control over his space and life. The particular context of Ali’s life, as someone who had fled violent conflict to seek asylum in the UK, was effaced by a mechanistic response to homelessness during the pandemic which was intent on making sure that homeless individuals were kept off of the streets and inside their rooms. Such vigilance was mobilised in order to protect wider ‘public health’ (indeed, underscoring removal of homeless people from the community of ‘the public’). The presence of security guards, from a private security firm subcontracted by the Local Authority, was as much a source of discontent for support workers as for clients. As one senior support worker remarked:

The security is hired by the council. We never had security prior to the hotels, it was something the council insisted on. We found it insulting actually, you know, we’ve only been doing this for years, and in the night beds too – the most volatile environment you can work in . . . We’re pushing to have the security gone, it’s just too many cooks. (Craig, senior support worker)

The inclusion of security surveillance introduced a fresh technology of regulation for service providers and service users, and for Ali engendered a sense of being hypervisibilised as a criminal or potentially criminal subject. This became all too much when one day, whilst walking towards the exit door to log out ahead of a walk to town, Ali overheard a member of the security staff referring to clients as ‘paedophiles’:

Yeah! They said: ‘We don’t give a damn about these people, they’re all paedophiles’. And then I said, ‘hang on, I’m not a paedophile, what are you talking about?’ And I just didn’t say to anybody, I wanted to get out as quickly as possible from that place.

### Joshua

When Joshua suddenly lost his job, it was the NRPF condition attached to his immigration status that blocked him from accessing the support necessary to avoid homelessness. In common with others interviewed, Joshua appeared ashamed that he was now in receipt of state support, and concerned that he might appear to be ‘exploiting’ the system:

I’m not expecting anything that is *charity* or anything.

I also agree that the system here, the government here, has to be tough on immigration. You don’t come into my house and mess up my house. You come decently and live decently – I strongly believe in that.

These unprompted statements illuminate Joshua’s need to justify his presence within support services *as a migrant*, a presence which left him feeling hypervisible as a potentially ‘undeserving’ (Shilliam, 2018) subject. Indeed, this reflects wider socio-political currents in the UK which construct migrants as motivated by opportunities to exploit the welfare state, and which therefore construct the welfare state as in need of protection from migrants (Tyler, 2013). This leaves individuals like Joshua internalising these attitudes and reflecting negatively upon their own receipt of support. Indeed, Joshua frequently positioned himself as ‘matter out of place’ in the homelessness service supporting him:

Now I’m being told once my papers have gone to the Home Office they will be taking me out of this place, put in some private home for homeless people. Of course, I don’t like to go and live in somebody’s house. I feel that I would be interfering with their privacy. However nice they are I don’t want to stand in their way. So that is something that is bothering me for the last few days. I was told about this last week. I don’t know what they’re going to tell me, the manager concerned is on leave . . . I’m tempted, to be very frank, [to] go back to the bus, put my feet somewhere, travel on the bus, because I don’t want to bother nobody.

Whilst Joshua was legally entitled to a room in a shared house, and would be living with other individuals experiencing homelessness, he felt like taking the room would be to encroach upon the ‘private’ space of the others he would be living with – so much so that he was considering returning to using the night buses. In the context of an increasingly hostile environment for migrants in the UK, welfare services are constructed as normatively white spaces, leaving migrants exposed as out of place, and questioning their own un/deservingness of support. Joshua’s unambiguous expressions of discomfort relating to his housing situation underscore his cultivated invisibility as embodied, habitual, and also sensorial.

## Conclusion: Cultivating invisibility

Drawing on the life stories of Albert, Joshua, Ali and Alexandru, this article seeks to attain a more nuanced understanding of migrant invisibility. We argue that the UK government’s Everyone In during the COVID-19 crisis provides a key moment through which to conceptualise this phenomenon. In contrast to research that views migrant invisibility as a *strategic* mode of action, deployed in response to the fear of state violence and/or deportation, we argue that cultivated invisibility is a habitual, embodied mode of practice created out of conditions of material scarcity and in the course of multiple engagements over time with racial capitalism’s various ‘faces of the state’. It involves blending into the crowd and/or staying on the move as a means of survival, but also results from uncomfortable experiences of coming forward in search of help. During Everyone In, there were unprecedented, and very welcome, offers of help. At the same time, migrants experiencing homelessness were hypervisibilised by various forms of surveillance, and felt stigmatised as ‘matter out of place’. Their uncomfortable experiences of being stigmatised in this way explains at least in part why they practised cultivated invisibility before the COVID-19 crisis. Thinking about the experiences of homeless migrants enables us to understand invisibility as intersectional, both produced and experienced along the lines of race, class and immigration status. As such, it is impossible to separate the effects of the hostile environment from the effects of austerity. In addition, we see invisibility as more than an effect of illegality, but rather as something that cuts across the experiences of both regular and irregular migrants. As individuals become increasingly invisibilised, through experiences such as precarious employment, job loss, homelessness, destitution and exclusion from access to the welfare state, exposure to illegality is increased and exacerbated. Nowhere is this clearer, or more devastating, than in the UK government’s 2021 announcement that rough sleeping will increasingly function as grounds for deportation within the UK border regime (Gov.uk, 2021). Our research situates this measure in a wider politics of vulnerability within which invisibility becomes a cause of illegalisation, just as much as a response to it.

## Notes

1. For Stephens and Stephenson (2016), the UK housing crisis must be understood in the wider context of austerity, and as part of a ‘radical reorientation’ of policy since 2010, through which successive governments have increased barriers to affordable housing for low income groups and withdrawn safety measures put in place to protect them. The National Housing Federation estimates that 8.4 million people in England alone are living in unaffordable or unsuitable accommodation, with 3.6 million living in overcrowded homes and 1.4 million in poor quality homes. The BBC’s (2020) Housing Briefing estimates that 1.2 million fewer homes have been built in the UK than required by the population, and that if current building rates stay the same it will take 15 years to close this gap.

2. No Recourse to Public Funds (NRPF) conditions apply to any person subject to immigration control under section 115 of the 1999 Immigration and Asylum Act. This applies to a whole host of immigration statuses including those related to student, spousal, visitor and family visas and asylum-seeker statuses (whether refused or in process), and other individuals with unresolved immigration statuses – also referred to as ‘undocumented’ migrants (see Legislation.gov.uk, 1999).

3. This name, and all of the others used in this article, are pseudonyms chosen by the respondents.
